# Prevalence and Risk Factors of Lower Limb Amputation in Patients with End-Stage Renal Failure on Dialysis: A Systematic Review

**DOI:** 10.1155/2016/4870749

**Published:** 2016-07-27

**Authors:** Rajit A. Gilhotra, Beverly T. Rodrigues, Venkat N. Vangaveti, Usman H. Malabu

**Affiliations:** School of Medicine and Dentistry, James Cook University, Townsville, QLD 4811, Australia

## Abstract

*Background.* Renal dialysis has recently been recognised as a risk factor for lower limb amputation (LLA). However, exact rates and associated risk factors for the LLA are incompletely understood.* Aim.* Prevalence and risk factors of LLA in end-stage renal failure (ESRF) subjects on renal dialysis were investigated from the existing literature.* Methods.* Published data on the subject were derived from MEDLINE, PubMed, and Google Scholar search of English language literature from January 1, 1980, to July 31, 2015, using designated key words.* Results.* Seventy studies were identified out of which 6 full-text published studies were included in this systematic review of which 5 included patients on haemodialysis alone and one included patients on both haemodialysis and peritoneal dialysis. The reported findings on prevalence of amputation in the renal failure on dialysis cohort ranged from 1.7% to 13.4%. Five out of the six studies identified diabetes as the leading risk factor for amputation in subjects with ESRF on renal dialysis. Other risk factors identified were high haemoglobin A1c, elevated c-reactive protein, and low serum albumin.* Conclusions.* This review demonstrates high rate of LLA in ESRF patients receiving dialysis therapy. It has also identified diabetes and markers of inflammation as risk factors of amputation in ESRF subjects on dialysis.

## 1. Introduction

End-stage renal failure, defined as nonreversible kidney damage requiring replacement therapy [1], is a recognised risk factor for peripheral artery disease leading to nonhealing ulcers and lower limb amputation (LLA) [2–6]. The aetiology of ESRF such as diabetes may be associated with complex vascular dysfunction and widespread organ involvement including cardiovascular and musculoskeletal systems, the two being the most important cause of morbidity and mortality in patients with ESRF on haemodialysis [1, 2, 7].

The prevalence of diabetes globally, as reported by the International Diabetes Federation in 2015, was 415 million (8.3% of the world's adult population) and is projected to alarmingly rise to 642 million by 2040, a significant rise over a small period of time [8, 9]. Diabetes is a leading cause of foot ulcer and renal failure amongst others [10]. In the case of foot ulcer, the combination of vascular insufficiency and local infection worsens the prognosis resulting in life threatening sepsis, LLA, and death [11–14]. Furthermore, the prevalence of foot complications in general is considerably higher in patients with diabetes and ESRF as compared to patients with diabetes without ESRF [5, 6, 13, 14]. It is known that LLA increases patient disability, decreases quality of life, and contributes to high morbidity, mortality, and health care costs [15–17] yet not much information on the extent of the problems is known worldwide.

Although various studies investigating the outcome of foot complications in patients with diabetes have been reported, prevalence of lower limb amputation in subjects on renal dialysis remains poorly recognised [6, 18]. Furthermore, clinical and biochemical features accounting for the high rate of LLA in subjects on renal dialysis are inconclusive. This systematic review aimed to critically appraise published studies which have assessed LLA as an outcome in patients with ESRF on dialysis and to determine prevalence and risk factors associated with LLA in the study population.

## 2. Methods

### 2.1. Protocol and Focus

This systematic review was performed with the standardised written protocol that followed the Preferred Reporting Items for Systematic Reviews and Meta-Analyses (PRISMA) guidelines [25]. The review focuses on studies which assessed the prevalence and identified risk factors for lower limb amputation in patients who have received renal dialysis.

### 2.2. Search Criteria

A search strategy was formulated to identify studies in which LLA was assessed in patients with ESRF on dialysis. Databases from MEDLINE/PubMed (US National Library of Medicine, Bethesda, MD, USA) and Google Scholar (Google, Mountain View, CA, USA) were searched from January 1, 1980, to July 31, 2015. Keyword sets combined “diabetes” or “diabetic ulcer” or “diabetic foot” and “amputation” and “renal dialysis”.

### 2.3. Eligibility Criteria

To be eligible, studies were required to focus on amputation as an outcome in ESRF patients on renal dialysis. For inclusion, studies had to be published before July 31, 2015. Publications were restricted to human studies and those published in English. There was no restriction on study size. For the inclusion of publications, the studies needed to be full articles which investigated patients on haemodialysis and/or peritoneal dialysis as a cohort and required recording of the prevalence and/or the risk factors associated with amputation as an outcome in this cohort.

Publications were excluded from this systematic review if they were review articles, looked at traumatic/neoplastic, only upper extremity/penile amputation as outcomes, compared prognosis of different interventions, and included only a subgroup of dialysis patients in the study (such as patients with diabetes on renal dialysis and patients on renal dialysis who had peripheral artery bypass).

### 2.4. Data Extraction

Data from the identified studies was extracted by one author. Any uncertainty was resolved by discussion between authors. Data extraction from eligible literature included information regarding geographical location, sample size, mean age, percentage of male patients, mean time on dialysis, diabetes mellitus, smoking history, hypertension, coronary artery disease, amputation, and risk factors (RR, OR, HR, RH, *p* values, and confidence intervals). Data was transcribed into an excel data collection sheet.

## 3. Results

### 3.1. Search Results

**Figure 1:**
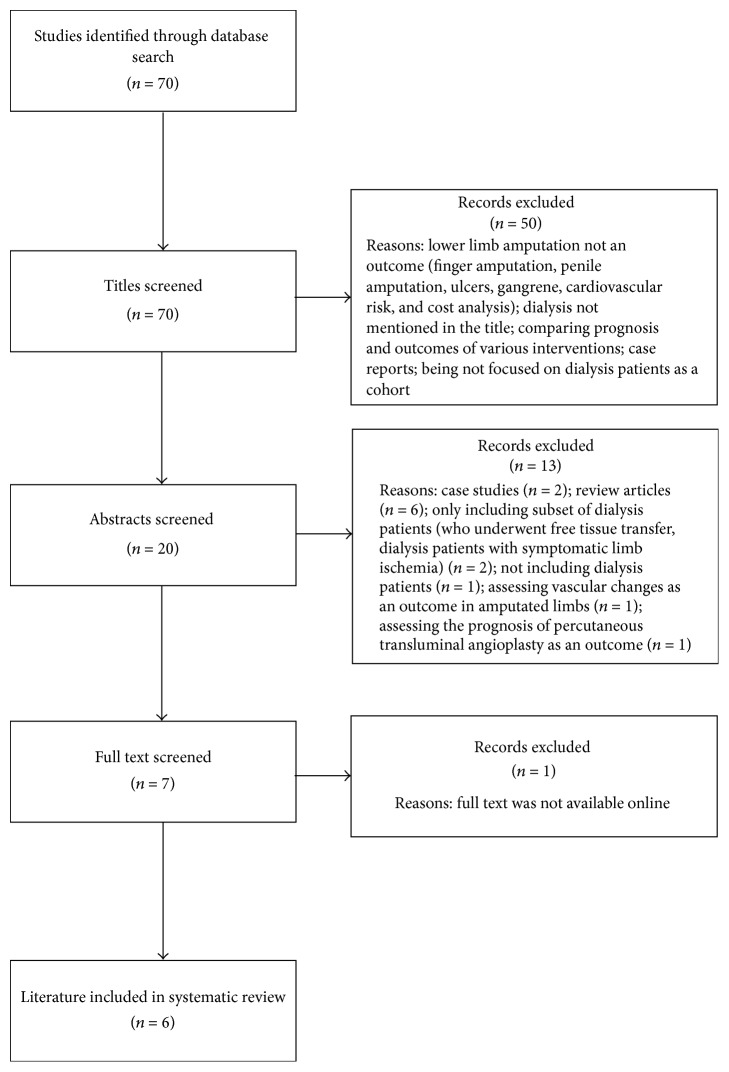
Flow diagram to illustrate the studies identified for this review.

Seventy published studies were identified as per the abovementioned search criteria (Figure 1). Fifty studies were excluded due to various reasons such as lack of investigating lower limb amputation as an outcome, dialysis/renal replacement/renal replacement therapy/end-stage renal failure/renal failure/end-stage kidney disease/kidney failure not being mentioned in the title, and comparing prognosis and outcomes of various pharmacological and surgical interventions. Abstracts were critically screened for the 20 remaining studies out of which another 14 were excluded due to being review articles (*n* = 6); being case studies (*n* = 2); only including a subset of dialysis patients (*n* = 2); not including dialysis patients (*n* = 1); assessing vascular changes in amputated limbs as an outcome (*n* = 1); assessing prognosis of percutaneous transluminal angioplasty as an outcome (*n* = 1); inability to access full text (*n* = 1). In all, 6 full-text publications were reviewed as detailed in Figure 1.

### 3.2. Characteristics of Included Studies and Subjects

Five out of the six studies included patients on haemodialysis [19–23] and one study included both haemodialysis and peritoneal dialysis patients [24]. There was no study that only included peritoneal dialysis patients.

As for the outcomes, two of the studies investigated only amputation in particular [19, 22] and the remaining four explored other outcomes as well such as foot problems, peripheral vascular disease related procedures, myocardial infarction, and mortality [20, 21, 23, 24]. The data related to these outcomes was presented separately in all these studies; therefore, it was possible to assess data specific to amputation by itself. There was marked variation in sample size between studies. All studies included at least 100 subjects (Table 1). Two studies included between 100 and 500 patients (*n* = 271, *n* = 232) [20, 21], three studies had between 1000 and 5000 subjects (*n* = 1513, *n* = 1041, and *n* = 3272) [19, 23, 24], and one study had 29838 subjects [22]. Most publications sourced participants from hospital or dialysis clinics, apart from one study that was conducted on patients from a nursing home setting [21]. Three of the studies recruited participants from single centres [20, 21, 23] and three from multiple centres [19, 22, 24]. Populations examined also varied between studies. Three studies focused on American patients [19, 21, 24]. Single studies recruited patients from Japan [23] and Canada [20]. One study included patients from around the world including 12 countries [22]. Three studies extracted data from larger studies such as the Choices for Healthy Outcomes in Caring for End-Stage Renal Disease (CHOICE) [24], Dialysis Outcomes and Practice Patterns Study (DOPPS) [22], and the ESRF Core Indicator/Clinical Performance Measure (CPM) Project [19].

All the studies were consistent in defining their inclusion criteria of patients on dialysis therapy; they all identified that patients with ESRF were placed on renal dialysis as a form of renal replacement therapy, that being either haemodialysis or peritoneal dialysis. The studies defined the endpoint being development of amputation, which could have been performed when uncontrollable limb infection existed even after undergoing revascularization and/or medical treatment. One publication specifically defined nontraumatic lower limb amputation and excluded digital amputation as one of the primary outcomes that were investigated [24]. Only one study specifically defined major amputation as above-the-knee amputation [23].

Five of the studies were longitudinal cohort studies, out of which four were retrospectively designed [19, 21, 22, 24] and one of each of prospective longitudinal format [23] and observational case-control study [20], respectively. Although all the studies were investigated for comorbidities in the participant population, there were some variations in the different studies. The prevalence of comorbidities also varied from study to study; for example, only 4.4% [23] of participants in one study had coronary artery disease compared to 54% in another [21]. All the retrospective studies collected their data via databases or medical chart review. Locking-Cusolito and colleagues used a foot assessment instrument which was used in clinics to collect data from patients attending their clinic [20]. The prospective study had a follow-up time period of 96 months over which the data was collected via direct patient contact, medical charts, or telephonic interview.

### 3.3. Amputation in Subjects on Renal Dialysis

The reported finding on prevalence of amputation in patients with renal failure on renal dialysis was 1.7% to 13.4% (Table 1). Five out of the six studies that were included in the systematic review identified presence of diabetes mellitus as the leading risk factor for amputation (*p* < 0.05) [19, 20, 22–24]. Although all the studies used different ratios to present their findings, there were two studies which used hazard ratios (HR). Ishii and colleagues and Speckman and colleagues reported that diabetic renal failure patients had a higher risk of getting amputation as compared to their counterparts without diabetes, HR 5.29, 95% CI 2–13.9, *p* = 0.0008, and HR 7.4, 95% CI 4.1–13.5, *p* < 0.001, respectively. On the other hand, Reddy and colleagues investigated the association of duration of renal replacement therapy and amputation as an outcome. They found that a higher percentage (19%) of established haemodialysis patients (dialysis for ≥12 months) had to have amputation as compared to new haemodialysis patients (8%) (dialysis for ≤3 months) (*p* = 0.01) [21].

### 3.4. Risk Factors of Amputation in Subjects on Renal Dialysis

Detailed risk factors are shown in Table 2. Diabetes was reported as a leading risk factor associated with amputation in renal dialysis patients in 5 out of the 6 studies. Two studies compared diabetic and nondiabetic subjects on dialysis showing higher rate of amputation amongst diabetics as shown in Table 3.

Other amputation risk factors reported in subjects on renal dialysis included longer duration of dialysis therapy, HbA1c levels, c-reactive protein (CRP), and low serum albumin. In patients with diabetes, a higher HbA1c level after commencement of dialysis was found to be statistically significant as a risk factor for amputation [20, 23]. Ishii and colleagues established that all patients requiring major amputation (*n* = 7) were found to fall in the highest HbA1c quartile (HbA1c > 6.8%). Overall, 6.1% of all amputees had an HbA1c > 6.8% as compared to only 0.9% of amputees with HbA1c < 5.4%. The association of a higher HbA1c and amputation was also found to be statistically significant: HbA1c > 6.8%, HR 2.99, 95% CI 1.17–7.7, *p* = 0.023. A higher CRP level, as a marker of inflammation, was found to be related to amputation by Ishii and colleagues, HR 1.01, 95% CI 1–1.02, *p* = 0.047, and by Plantinga and colleagues showing relative hazard (RH) 2.7, 95% CI 1.72–4.23, *p* < 0.05 [24]. Subjects on renal dialysis with a low mean serum albumin (<3.3 g/dL) were also found to be at risk of amputation by Speckman and colleagues: HR 1.8, 95% CI 1.1–3, *p* < 0.05. If these subjects had diabetes, the risk was further increased: HR 3.8, 95% CI 1.1–12.5, *p* < 0.05. In the same study, Plantinga et al. reported increased albumin levels were associated with significantly decreased risk of amputation: RH 0.63, 95% CI 0.42–0.94, *p* < 0.05 [24].

## 4. Discussion

This review highlights the scarcity of literature which investigates the prevalence and risk factors of amputation in patients with ESRF on renal dialysis. It revealed 1.7% to 13.4% as prevalence of LLA in patients receiving renal dialysis therapy [20, 23]. This showed a wide variation in amputation between studies and countries. For instance, study from Japan reported lower prevalence of LLA in subjects on renal dialysis compared to higher rate in USA and Canada. This variation could suggest a better outcome amongst the Japanese cohort. This is in keeping with the finding of lower rate of coronary artery disease in the population, which is identified as an important risk factor for LLA [1, 2, 7]. It could also suggest that North Americans have a lower threshold for LLA. Other reasons for the wide variation in prevalence might be due to multiple factors. For instance, Combe and colleagues conducted a large scale multinational, multicentre study which included a large number of subjects (29838) and investigated various associated risk factors such as age, ethnicity, calciphylaxis, retinopathy, and neuropathy [22]. However, the methodology used to collect the data did not differentiate between types of amputation. Though this study presented an overall worldwide 6% prevalence of amputation in renal dialysis patients, it may have been an overestimation. Plantinga and colleagues on the other hand excluded digital amputation from their data collection; this might have resulted in an underestimation of all amputations in renal dialysis patients [24]. Nevertheless, these findings warrant further studies.

Interestingly, we have identified presence of diabetes as an important single risk factor for LLA, with up to 26.5% of patients with diabetes receiving renal dialysis having amputation [20]. Renal dialysis has been found to increase the risk of foot ulcer in patients with diabetes. This significant temporal association is suggested by a 3 times higher risk of having foot ulcer in the first year of starting dialysis therapy and an almost 32 times higher risk for major amputation in the first year of starting dialysis therapy [26–28]. This risk is further increased in the presence of elevated CRP [23, 24] and low serum albumin [19, 24] as well as high HbA1c level [20, 23] at the onset of dialysis in agreement with recent reports [27, 28], suggesting that acute phase reactants and poor control of diabetes worsen the prognosis further. These findings were suggested from studies which were limited to patients on haemodialysis [19–23]. On the other hand, the only study which included both peritoneal dialysis and haemodialysis [24] reported a high prevalence of limb amputation of 13%; however, the authors did not provide a comparison between patients receiving haemodialysis and those on peritoneal dialysis.

Although studies have shown that peripheral arterial disease is common amongst patients on chronic dialysis therapy, the indication for bypass surgery in these patients is still a controversial topic. This is due to the high morbidity and mortality rates of these patients. Even though PTA is considered a possible procedure to treat critical limb ischemia, there is limited literature available on the prognosis of this intervention. In a 2-year follow-up prospective study on haemodialysis patients with diabetic foot wounds who were treated with PTA and minor amputation, it was found that only 41.9% of patients survived without reamputation with a 2-year postoperative mortality rate of 38.7% [29]. This study had a small sample size of 31 haemodialysis patients; hence, it is important to explore the prognosis of PTA as treatment and prevention of amputation by doing more studies with a larger sample size. Other aspects of the review include associations between surgical procedures for vascular access and amputation. In patients with and without diabetes, both variables were increased after initiation of haemodialysis therapy. Amputations, which are potentially preventable, were associated with significant mortality amongst haemodialysis patients [30]. Thus, early interventions at primary care level have been found to lower the rate of foot complications and amputation, further stressing the need for multidisciplinary care in order to prevent adverse outcome in this group of population [31].

## 5. Conclusion

This review has demonstrated the high rate of lower limb amputation in patients with ESRF receiving dialysis therapy. It has also identified the risk factors associated with an adverse outcome, the chief amongst them being presence of diabetes. Other identified risk factors include hypoalbuminemia and elevated CRP. Thus, with early intervention in diabetic subjects, many limbs would likely be salvaged and the overall rate of adverse outcome from LLA would eventually be lowered. However, there is still a limited literature on preventative programs for LLA in subjects on renal dialysis. Furthermore, association between peritoneal dialysis and LLA is scanty as most studies were conducted on haemodialysis subjects. More studies are needed to further characterise findings in these areas.

## Figures and Tables

**Table 1:** Characteristics of studies that assessed amputation in subjects on renal dialysis.

Author	Country	Total cases	Mean age (y)	Male (%)	Time on dialysis	DM	Smoking	HTN	CAD	Amputation (%)
Speckman et al., 2004 [19]	USA	3272 HD	—	53.4%	—	1751 (53.5%)	—	—	1711 (52.3%)	116 (4%)
Locking-Cusolito et al., 2005 [20]	Canada	232 HD	65.1	130 (56%)	—	98 (42.2%)	—	75%	50%	31 (13.4%)
Reddy et al., 2007 [21]	USA	271 HD	70.5 ± 12	127 (47%)	18 ± 27 (m)	176 (65%)	37 (14%)	244 (90%)	146 (54%)	34 (13%)
Combe et al., 2009 [22]	Multinational	29838 HD	61.3 ± 15	57.7%	3.3 ± 5 (y)	11129	17.7%	77.2%	41.6%	6%
Ishii et al., 2012 [23]	Japan	1513 HD	63 ± 13.5	66.7%	—	739	22.6%	74.05%	4.4%	26 (1.7%)
Plantinga et al., 2009 [24]	USA	1041 (767 HD, 274 PD)	57.9 ± 15	54.2%	—	54%	60.6%	—	24.5%	136 (13%)

HTN, hypertension; CAD, coronary artery disease; DM, diabetes mellitus; HD, haemodialysis; PD, peritoneal dialysis; (y), years; (m), months; USA, Unites States of America.

**Table 2:** Summary of studies assessing risk factors of amputation in subjects on renal dialysis.

Author	Risk factors for amputation in all dialysis patients
Speckman et al., 2004 [19]	MUR < 58.5%: HR 2.4 (CI 1.4–4.2), *p* < 0.01 CVD: HR 2.4 (CI 1.6–3.7), *p* < 0.001

Combe et al., 2009 [22]	DM: AOR 5.55 (CI 4.63–6.64), *p* < 0.001 Men: AOR 1.82 (CI 1.61–2.06), *p* < 0.001 Blacks: AOR 1.45 (CI 1.28–1.65), *p* < 0.001 Smoking: AOR 1.22 (CI 1.03–1.43), *p* < 0.001 Retinopathy: AOR 1.78 (CI 1.55–2.04), *p* < 0.001 Peripheral neuropathy: AOR 2.25 (CI 1.98–2.56), *p* < 0.001 Calciphylaxis: AOR 2.37 (CI 1.63–3.45), *p* < 0.001

Plantinga et al., 2009 [24]	DM: RH 4.33 (CI 2.98–6.30), *p* < 0.05 PVD: RH 1.59 (CI 1.17–2.15), *p* < 0.05 CRP: RH 2.7 (CI 1.72–4.23), *p* < 0.05 Albumin < 3.3 g/dL: RH 0.63 (CI 0.42–0.94), *p* < 0.05

Locking-Cusolito et al., 2005 [20]	DM: AOR 10.17 (CI 3.7–27.7), *p* < 0.001

Ishii et al., 2012 [23]	DM: HR 5.29 (CI 2–13.9), *p* = 0.0008 CRP: HR 1.01 (CI 1–1.02), *p* < 0.05

Reddy et al., 2007 [21]	Dialysis > 12 months: AOR 2.74 (CI 1.22–6.16), *p* < 0.05

DM, diabetes mellitus; HR, hazard ratio; RH, relative hazard; AOR, adjusted odds ratio; PVD, peripheral vascular disease; CVD, cardiovascular disease; MUR, mean urea reduction; CI: 95% confidence interval.

**Table 3:** Summary of studies assessing risk factors of amputation in diabetic and nondiabetic patients on dialysis.

Author	Total patients with DM	DM patients having amputation	Risk factors for amputation in patients with DM	Total non-DM patients	Non-DM patients having amputation	Risk factors for amputation in patients without DM
Speckman et al., 2004 [19]	1751	104 (6%)	CVD: HR 1.7 (CI 1.1–2.6), *p* < 0.05 MUR < 58.5%: HR 2.6 (CI 1.4–4.8), *p* < 0.01	1469	12 (1%)	Increasing age: HR 1.09 (CI 1.02–1.2), *p* < 0.01 Mean serum albumin < 3.5 g/dL: HR 3.8 (CI 1.1–12.5), *p* < 0.01

Combe et al., 2009 [22]	11129	14.2%	Peripheral neuropathy: AOR 2.23 (CI 1.94–2.56), *p* < 0.001 Calciphylaxis: AOR 2.02 (CI 1.32–3.1), *p* < 0.001 Men: AOR 1.69 (CI 1.48–1.94), *p* < 0.001	18203	1.60%	Calciphylaxis: AOR 3.41 (CI 1.74–6.7), *p* < 0.001 Men: AOR 2.56 (CI 1.86–3.51), *p* < 0.001 Blacks: AOR 1.88 (CI 1.23–2.86), *p* < 0.001 Smoking: AOR 1.62 (CI 1.11–2.37), *p* < 0.001

DM, diabetes mellitus; HR, hazard ratio; RH, relative hazard; AOR, adjusted odds ratio; CVD, cardiovascular disease; MUR, mean urea reduction; CI: 95% confidence interval.
